# Dental Fear and Anxiety in Children and Adolescents: Qualitative Study Using YouTube

**DOI:** 10.2196/jmir.2290

**Published:** 2013-02-22

**Authors:** Xiaoli Gao, SH Hamzah, Cynthia Kar Yung Yiu, Colman McGrath, Nigel M King

**Affiliations:** ^1^Dental Public HealthFaculty of DentistryThe University of Hong KongHong Kong SARChina; ^2^Paediatric Dentistry and OrthodonticsFaculty of DentistryThe University of Hong KongHong Kong SARChina; ^3^Faculty of DentistryUniversiti Teknologi MaraShah AlamMalaysia; ^4^Paediatric DentistryFaculty of Medicine, Dentistry and Health SciencesUniversity of Western AustraliaPerthAustralia

**Keywords:** dental fear, dental anxiety, children, adolescents, qualitative research, Internet social media

## Abstract

**Background:**

Dental fear and anxiety (DFA) refers to the fear of and anxiety towards going to the dentist. It exists in a considerable proportion of children and adolescents and is a major dilemma in pediatric dental practice. As an Internet social medium with increasing popularity, the video-sharing website YouTube offers a useful data source for understanding health behaviors and perceptions of the public.

**Objective:**

Using YouTube as a platform, this qualitative study aimed to examine the manifestations, impacts, and origins of DFA in children and adolescents from the public’s perspective.

**Methods:**

To retrieve relevant information, we searched YouTube using the keywords “dental fear”, “dental anxiety”, and “dental phobia”. Videos in English expressing a layperson’s views or experience on children’s or adolescent’s DFA were selected for this study. A video was excluded if it had poor audiovisual quality, was irrelevant, was pure advertisement or entertainment, or contained only the views of professionals. After the screen, we transcribed 27 videos involving 32 children and adolescents, which were reviewed by a panel of 3 investigators, including a layperson with no formal dental training. Inductive thematic analysis was applied for coding and interpreting the data.

**Results:**

The videos revealed multiple manifestations and impacts of DFA, including immediate physical reactions (eg, crying, screaming, and shivering), psychological responses (eg, worry, upset, panic, helplessness, insecurity, resentment, and hatred), and uncooperativeness in dental treatment. Testimonials from children, adolescents, and their parents suggested diverse origins of DFA, namely personal experience (eg, irregular dental visits and influence of parents or peers), dentists and dental auxiliaries (eg, bad manner, lack of clinical skills, and improper work ethic), dental settings (eg, dental chair and sounds), and dental procedures (eg, injections, pain, discomfort, and aesthetic concerns).

**Conclusions:**

This qualitative study suggests that DFA in children and adolescents has multifaceted manifestations, impacts, and origins, some of which only became apparent when using Internet social media. Our findings support the value of infodemiological studies using Internet social media to gain a better understanding of health issues.

## Introduction

Fear of and anxiety towards going to dentists (ie, dental fear and anxiety, DFA) are major problems for a sizeable proportion of children and adolescents. The prevalence of DFA in children and adolescents ranges from 5-20% in various countries, with some cases being considered to be dental phobia (severe DFA) [1-3]. Children and adolescents with DFA are often uncooperative during dental visits, thus rendering treatment difficult or impossible [3]. Such behavior compromises the treatment outcome, creates occupational stress among dental staff, and is often a cause of discord between dental professionals and patients or their parents [4]. Fearful children and adolescents may try every possible means to avoid or delay treatment, resulting in deterioration of their oral health [4-6]. Beyond its impacts on dental care, DFA may also cause sleep disorders, affect one’s daily life [7] and have a negative impact on one’s psychosocial functioning [8]. DFA acquired in childhood may persist to adulthood and is a significant predictor for avoidance of dental visits in adulthood [9,10]. This pinpoints childhood as a critical stage for preventing and intercepting DFA, thereby assisting people to protect their oral health in the long term.

Previous studies into DFA draw predominantly upon quantitative instruments such as questionnaires and psychometric scales [3]. The development of these instruments, however, is largely based on professionals’ presumptions and thus may not capture the whole spectrum of respondents’ perceptions and views. Moreover, quantitative methods that focus on generating statistics and testing hypotheses may not be able to uncover complex mechanisms [11]. Qualitative research approach is therefore considered an important complement to quantitative methods, especially for gathering in-depth information on human behavior and reasons for such behavior [11]. Although qualitative studies do not aim to provide data that are statistically extrapolatable to a wide population, they can delineate a wide range of views and experiences in peoples’ own words and a rich context [11].

Currently, there is a paucity of research employing qualitative methodologies for understanding DFA, although a few qualitative studies have produced some enlightening findings [12-14]. Abrahamsson and coworkers, through thematized in-depth interviews with 18 patients, showed that individual vulnerability and traumatic dental care experiences caused dental fear in adult patients, who were often caught in a vicious cycle of fear and negative expectations about treatment [12]. They also found that several psychological and social factors such as self-respect, well-being, avoidance, readiness to act, and ambivalence in coping, determined how adult patients coped with their fear and how dental fear affects their daily lives [13]. Through semi-structured interviews with mothers of 14 children who were uncooperative during dental treatment, 3 themes explaining children’s refusal to submit to dental treatment were identified. These included the origins of child behavior, caregivers’ attitudes, and the culture of resistance [14]. The findings of these studies suggest that qualitative analysis is a useful method to further our understanding of DFA.

On the other hand, recent medical studies have illustrated the potential of utilizing public uploads on Internet social media, such as YouTube, as a valuable source of qualitative data to understand health behaviors and perceptions [15-18]. For instance, through an analysis of 35 YouTube videos, a study has reported the personal narratives of cancer survivors and enriched our understandings on the psychological impact of cancer diagnosis on patients’ personal and family lives [17]. The findings help professionals communicate with patients and their families more effectively and provide better care to cancer patients. The potential of Internet social media in dental research is however, hardly explored. Recently, Knösel and coworkers reported an interesting work, where they systematically assessed educational videos on YouTube related to dentistry [19]. Their analysis suggested the potential value of YouTube in dental education and its role in shaping public opinion about the dental profession.

YouTube is an online video-sharing website founded in 2005. It records more than 3 billion views a day and 800 million users each month [20]. YouTube offers an unrestricted environment for the public to share their stories and express their feelings instantaneously and freely. It helps individuals to discuss sensitive issues easily or to venture opinions without fear of embarrassment or negative judgement, which is often a concern in face-to-face interviews [21]. Theoretically, the candid in-depth testimonials and reports on YouTube could be useful data sources for investigating DFA. The personal narratives and original sharing uploaded spontaneously by patients and the public to YouTube provide a rich context to our existing knowledge on DFA. In addition, some novel or relatively neglected themes may emerge and thus deepen our understanding of DFA. This study aimed to profile the manifestations, impacts, and origins of DFA in children and adolescents from the public’s perspective using a qualitative research approach and YouTube as a platform.

## Methods

### Video Search and Screening

YouTube videos were searched using the 3 keywords “dental fear”, “dental anxiety”, and “dental phobia”. Since uploads to Internet social media turnover frequently, we chose 3 consecutive days in August 2010 and finished the search in this fixed period.

All of the identified videos were screened for eligibility for this study. A video was included in the study if it was in English and expressed views or experiences of a layperson with no formal dental training on any aspect of DFA. A video was excluded if: (1) it was not related to DFA, (2) it was purely an advertisement, (3) it was purely for entertainment (eg, comedy), (4) it contained only the views of dental professionals, (5) it was in a language other than English, or (6) its production quality was unacceptably poor such that the meaning of the speech or conversation could not be discerned. The screening was performed by one of the authors (HSH). When there was any doubt or ambiguity, discussions took place among authors until a consensus was reached.

Only videos concerning DFA in children and adolescents were included in this study. A further screen on the videos was made based on the age of the person experiencing DFA, not the age of the informant (eg, a video in which a mother talked about her young daughter’s DFA was classified as a video on children’s DFA). In 5 videos, the age of the children or adolescents was disclosed by themselves or their parents during the conversation, or by the video authors in their video descriptions. In other videos where age was not explicitly disclosed, since it was impossible to identify the exact age of the subjects, the judgement of age was mainly based on visual and verbal clues (ie, appearance, behaviors, and level of speech development) [15].

### Transcription and Content Analysis

The selected videos were transcribed verbatim. Non-verbal expressions such as facial expressions and body postures were also described. A panel of 3 members consisting of a pediatric dentist, a behavioral scientist/public health practitioner, and a layperson with no dental background, read through the transcripts and watched each video carefully to ensure that the context was precisely understood and documented.

Thematic content analysis [11] was applied. Transcripts were analyzed by means of line-by-line coding manually. No data analysis software was used. Themes were developed mainly through an inductive method (ie, as they emerged from the data). The key elements that were relevant to the area of inquiry were identified and labelled concretely by using either the informant’s words (in vivo codes) or the words and concepts of the researchers’ disciplines (in vitro codes). This process of open coding led to a clustering of substantive codes with similar content into themes, which were subsequently grouped and organized under analytical categories [14].

All analysis was done through discussions among the 3 review panel members. The members strived to avoid being governed by their own pre-structured understanding and to maintain a self-reflective attitude to ways in which the review process could be influenced. To ensure reflexivity, competing explanations and alternative interpretations were taken into consideration throughout the analysis. During theme development and coding, any ideas, preliminary assumptions, and theoretical reflections were noted and considered in the analysis. A certain degree of disagreement existed among panel members in coding of approximately 6% of the total contents. Discussions took place whenever there were disagreements until consensus was reached.

To characterize the key elements of each thematic category, the overall descriptions of all the videos involved were presented. Original quotes, verbatim excerpts, or illustrative examples drawn from the videos were provided whenever possible to facilitate a comprehensive understanding of the themes.

## Results

### Videos and Statistics

**Figure 1:**
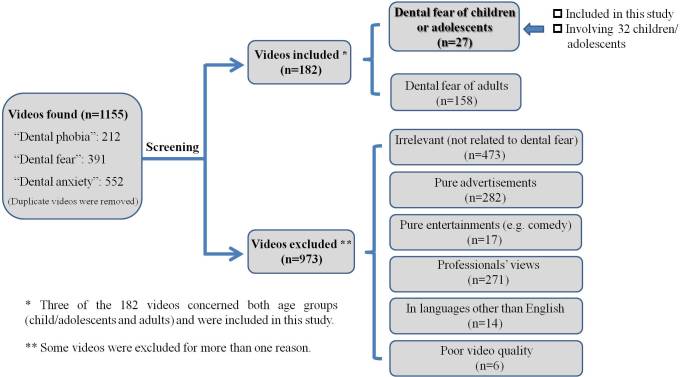
Video search and selection results.

A total of 1155 videos were retrieved under the 3 keywords (Figure 1). After screening, 182 videos were found concerning the public’s views or experiences on DFA. Among these, 27 videos were about DFA of 32 children or adolescents (17 males and 15 females) and were analyzed in this report. These included 3 videos concerning both age groups (children/adolescents and adults).

Most videos were uploaded from the United States. Two thirds of the videos were about DFA of children, with the remaining third on DFA of adolescents (Table 1). In over three quarters (21/27, 78%) of the videos, children or adolescents shared their own stories and feelings, whereas in the remaining videos, parents were the proxy informants. The duration of the videos ranged from about half a minute to 10 minutes. Half of the videos lasted 1 to 2 minutes. Most (25/27, 93%) of the videos were uploaded in the past 2 years (2009 and 2010). About half of the videos had been viewed hundreds to thousands of times. Five videos were very popular, with more than 10,000 views each.

**Table 1:** Video statistics (N=27).

		n (%)
**Subject concerned**		
	Child	18 (67)
	Adolescent	9 (33)
**Informant(s)**		
	Self (child/adolescent)	21 (78)
	Parent(s)	4 (15)
	Both	2 (7)
**Video duration**		
	<1 min	4 (15)
	1-2 mins	13 (48)
	3-8 mins	6 (22)
	9-10 mins	4 (15)
**Time being uploaded**		
	Year 2005-2008	2 (7)
	Year 2009	12 (44)
	Year 2010 (till August)	13 (48)
**Country of origin**		
	United States	20 (74)
	Australia	3 (11)
	UK	2 (7)
	Afghanistan	1 (4)
	Unknown	1 (4)
**Number of views**		
	<100	9 (33)
	100-1000	5 (19)
	1001-10,000	8 (30)
	10,001-100,000	4 (15)
	>100,000	1 (4)
**Number of rating**		
	0	18 (67)
	1-10	6 (22)
	11-100	3 (11)
**Number being selected as favourite** ^**a**^		
	0	18 (67)
	1-10	6 (22)
	11-100	3 (11)

^a^If a video was selected by a user as “favourite”, the user can keep track of the video from within his/her own account and channel. To a user, his/her favourite videos appear as a special playlist.

### Manifestations and Impacts of Dental Fear and Anxiety

Themes emerged from the videos on the manifestations and impacts of DFA are summarized in Table 2. Each theme was supported by representative quotes and keywords and was organized into 1 of the 3 analytical categories.

**Table 2:** Manifestations and impacts of DFA on children and adolescents.

Analytical categories	Themes	Quotes/keywords
Immediate physical reactions	Cry, scream, shiver	Not applicable
Psychological responses	Worry, upset, panic	“*What if they drill a hole in my teeth?”* [video 15] “*It breaks bone down.”* [video 23] “*I dreaded, you know, that procedure.”* [video 18] “*Total anxiety attack and it was terrible.”*[video 24]
	Helplessness, insecurity	“*I was scared to have them [wisdom teeth] removed. I was afraid to turn 18.”* [video 18]
	Resentment, hatred	“*I absolutely hated the dentist. I hated going there.”* [video 18]
Uncooperativeness	Refuse to sit in dental chair	Not applicable
	Refuse to open mouth	Not applicable
	Refuse visiting dentist	Parent has to *“fight”* with children for them to go to clinic. [video 16]

#### 1. Immediate Physical Reactions

To children and adolescents, a visit to the dentist may represent a tremendous challenge. Often seen in the videos were their immediate physical reactions, such as crying piteously, screaming forcefully, and shivering uncontrollably.

#### 2. Psychological Responses

Psychological responses to DFA appeared as an amalgamation of worry, upset, panic, feeling of helplessness, insecurity, resentment, and hatred towards dentists. In a few videos, some teenagers explicitly expressed worry and panic towards dental treatments.

I’m still nervous about getting my braces. I’m afraid I am going to be choked like this. What if they drill a hole in my teeth

video 15

The needle, [I] completely have phobia of needle and I freak out. Total anxiety attack and it was terrible

video 24

Removal of wisdom teeth was a nightmare to some adolescents who had undergone or were to undergo the procedure. The night before the scheduled dental appointment, a teenage girl, shaking and smoking in front of the camera, shared her many concerns. She could not stop worrying about the possibility of *“*break[ing] bone down” during the surgery and that both of her cheeks would be swollen after the surgery [video 23].

A video featured a teenage boy who was overwhelmed by a strong sense of helplessness and insecurity after hearing the terrible stories of his close friends who had undergone wisdom tooth extraction. The fear penetrated so deeply inside him that ultimately he declared that he would rather not to turn 18 just to avoid the possible threat of wisdom tooth extraction. This fear, together with his sympathy towards his friends, gradually fermented into hatred towards dentists.

I was scared to have them removed. I was afraid to turn 18. I absolutely hated the dentist. I hated going there. And I dreaded, you know, the procedure.

video 18

#### 3. Uncooperativeness

Fearful pediatric patients often refused to sit in the dental chair or open their mouths for oral examination. Parents and dental teams have to struggle to convince or encourage them to cooperate. A mother expressed that her daughter had at last conquered her fear of the dentist, but before that she had to “fight” with her daughter to get the daughter to go to the clinic [video 16].

### Origins of Dental Fear and Anxiety

Table 3 summarizes the themes of DFA that emerged from the videos. These fall into three major analytical categories.

**Table 3:** Origins of DFA of children and adolescents.

Analytical categories	Themes	Quotes/keywords
Self experience and parents/peers’ influence	Irregular dental attendance	“*He is rather stressed about going to the dentist. I’m not sure if it is because we are doing an actual visit or because we have not gone for a while.”* [video 16]
	Parents’ negative statement	Father told her *“The dentist would pull your teeth.”* [video 2]
	Horrible stories from friends	“*It was because I had a couple of friends who were having some massive dentistry done. And I felt terrible for them.”* [video 18]
Dentist/dental auxiliaries	Bad manner	Impatience
	Lack of clinical skills	“*It’s numb all the way up to my eye and over to the bottom of my chin. And they gave me... I don’t know, like six shots of numbing thing and like three of local anaesthetic gel. And I started crying in the chair… He cut my lip.”* [video 24]
	Improper work ethics	Lack of respect: “*So when I told [the dental auxiliary] that I want the hot pink one, she looked at me like I was a little cuckoo.”* [video 15] Unpunctuality: “*First, I waited and waited and waited and waited. It took like forever. Finally they called my name and I got to sit in the death chair.”* [video 15]
Dental setting/procedure	Dental chair and sound	“*Dentists’ chairs can be painful places…The sound alone is enough to send someone running.”* [video 16]
	Injection	“*The needle. [I] completely have phobia of needle.”* [video 24]
	Pain and discomfort	“*I’m going to be choked like this...It was really painful... The experience was dreadful overall.”* [video 15]
	Aesthetic concerns	“*I’m expecting the brace to break my appearance…If I go like this [grin], then it’ll break my appearance.”* [video 15]

#### 1. Self-experience and Influence of Parents or Peers

To some children, DFA may be caused by the irregularity of their dental visits, as illustrated by a testimonial from a mother who attributed her son’s fear to infrequent dental attendance.

He is rather stressed about going to the dentist. I’m not sure if it’s because we are doing an actual visit or because we have not gone for a while

video 16

Children’s fear can be instilled by careless words from parents. A video depicted the destructive behaviors of a young girl who refused to be examined in the clinic. After tiring persuasion and struggle, her mother explained to the dentist that she was acting uncooperatively and irrationally because her father had told her in a teasing tone that, “the dentist would pull your teeth” [video 2].

To adolescents, peers’ influence should not be underestimated. For a teenage boy who was extremely unwilling to seeing a dentist, what stroke fear into him initially was his friends’ story of their negative experience.

It was because I had a couple of friends who were having some massive dentistry done. And I felt terrible for them.

video 18

#### 2. Dentists and Dental Auxiliaries

Patients’ DFA may stem from professionals’ bad manners, as shown in an instance of a dentist treating a young girl. Without trying to ease the girl’s fear, the dentist rushed through the procedures, regardless of the child’s resistance. During the whole visit, the child could not follow the dentist’s instructions well. The dentist behaved impatiently and shone the lamp into the child’s eyes to force her to close her eyes. Here are the commands that were uttered by the dentist loudly, sternly, and impatiently.

Close your eyes. Keep it closed. Squeeze squeeze squeeze. Squeeze your eyes all the way. Hold them closed. You are opening. Close your eyes [shines the light]…Stay closed until I tell you to open again. Okay?...We won’t be able to do it.... Close your eyes…How can I…?

video 7

Professionals’ lack of clinical skills may be another cause of DFA. A teenage girl who had been treated by an incompetent dentist expressed mixed feelings of panic and anger.

It’s numb all the way up to my eye and over to the bottom of my chin. And they gave me... I don’t know, like six shots of numbing thing and like three of local anaesthetic gel. And I started crying in the chair. It was really painful… He cut my lip.

video 24

Improper work ethics of the dental team may exacerbate DFA. This happened to a teenage boy who did not feel treated respectfully by a dental auxiliary. While he chose the “hot pink” separator for orthodontic treatment, the dental surgery assistant looked at him as if he was “a little cuckoo” [video 15]. Such response indeed made him embarrassed and upset. In addition, unpunctuality could upset patients and worsen their anxiety before sitting in the dental chair, as expressed by an annoyed teenage boy.

First, I waited and waited and waited and waited. It took like forever. Finally they called my name and I got to sit in the death chair

video 15

#### 3. Dental Setting and Procedure

Besides the human factors mentioned above, the physical environment of the dental clinic could provoke fear to pediatric patients. Fear can be triggered by many elements in the clinic, from major dental equipments such as the dental chair, called by a teenage boy the “death chair” [video 15], to seemingly trivial details such as dripping sounds from the tap.

Dentists’ chairs can be painful places...The sound alone is enough to send someone running

video 16

In addition, certain dental procedures (eg, injections) were the main reasons for DFA of some pediatric patients.

It was really painful. The needle! [I] completely have phobia of needle and I freaked out

video 24

Similarly, the expectation of pain and discomfort (eg, choking) can lead to DFA.

I’m going to be choked like this...She twisted and turned all the braces and tucked them on my teeth. It was really painful...I was told that at 6 p.m. my braces will be really sore. She said the brace would hurt really badly for the next three days. So I think I’m going to fall in love with Mr. XXX [a pain relief pill] because I’m going to be taking that a lot. The experience was dreadful overall

video 15

Having an attractive appearance means a lot to adolescents. A teenage boy who was going to receive orthodontic treatment worried that the metal bars would make his appearance strange. His aesthetic concerns were the root of his anxiety towards the coming procedures.

I’m expecting the braces to break my appearance... For a whole year, I had an expander which basically expands my jaws… If I go like this [grins], then it will break my appearance

video 15

## Discussion

### Internet Social Media as Data Sources for Dental Research

The explosive growth of Internet social media has transformed the ways that individuals communicate with their surroundings and offers a unique opportunity for healthcare research. The vibrant information exchange through Internet social media is bidirectional. While the public can acquire large volumes of health messages readily [22-24], valuable data could be retrieved by health professionals from the Internet social media for research purposes [15-18]. The potential of Internet social media was however largely untapped in dental research. This study therefore addressed this gap and utilized YouTube to solicit the public’s views on an important dental issue—DFA in children and adolescents.

### Main Findings and Implications

Collectively, the videos revealed multifaceted manifestations, impacts, and origins of DFA among children and adolescents. Although immediate physical response and uncooperativeness were not unexpected, the nature and extent of the psychological impacts were striking. Facing the challenges of a dental visit, some children demonstrate externalizing behaviors such as tantrums, whereas some internalize the fear, which may lead to psychological or behavioral withdrawal, feelings of shame or inferiority, and low self-esteem [25]. The story of the teenage boy who would rather not turn 18 in order to avoid the possible threat of wisdom tooth removal was a vivid testimonial of the profound psychological impact of DFA. It exemplifies how DFA could impair children and adolescents’ outlook towards life; something dental professionals should not neglect.

This qualitative study has attached considerable and diverse real life stories to the heterogeneous origins of DFA through 3 pathways, namely direct conditioning via negative dental visit experience, vicarious learning from family and peers, and exposure to negative information [26]. A frequently quoted reason for the initiation and persistence of DFA was the expected pain and discomfort during invasive procedures, such as injections and extractions, and some contextual stimuli, such as syringes and dental chairs. While some of these fear-provoking factors may be alleviated through thoughtful planning of the treatment modality [27] and creative modifications of the physical settings of the clinic [28], some are not easy to change because there are no alternatives. Nonetheless, on an optimistic note, fear and anxiety is a multi-dimensional construct that consists of somatic, cognitive, and emotional elements [29]. The consequences of traumatic dental procedures depend on the context in which they occur. Previous research has suggested that pain inflicted by a dentist, who was perceived as caring, was likely to have less psychological impact than pain inflicted by a dentist who was cold and controlling [10]. This finding underscores the active role that dental professionals could play in conditioning and moderating patients’ response to invasive dental procedures.

Our analysis indeed highlights the importance of dental professionals’ manner, clinical skills, and work ethics in reducing DFA of pediatric patients. Children and adolescents lack maturity to fully manage their emotions and control their reactions [30]. Thus, they may require extra patience, which clinicians working with this age group should be prepared to offer. DFA might well arise from a perceived lack of respect, something dentists may tend to neglect when treating young patients. This finding echoes the results of a previous study that ranked dentists’ attitudes and comments as one of the most fear-stimulating factors, amidst invasive procedures such as extractions, drilling, and injections [31]. Our findings also highlighted the necessity of adhering to the original appointment time. During prolonged waiting, many elements in the clinic may trigger patients’ fear, which can accumulate to an unbearable level. In cases the dentist is unable to treat a patient on time, dental auxiliaries should introduce the patient into the world of dentistry, build a sense of closeness with the patient, and prepare the patient for the upcoming dental procedures. Engaging patients in these activities could avoid the escalation of their DFA during the waiting period.

To assist children and adolescents to experience success in managing their DFA, a partnership between parents and professionals is highly advocated [14]. Nevertheless, parents often feel powerless in managing their children’s DFA and blame the negative dental treatment [14], while dentists usually have a different frame of reference and tend to put the blame on parental factors, such as upbringing [32]. There is no doubt that, to children and adolescents, reactions from parents often craft their ways in manipulating their environments and regulating their behavior and are certainly one of the most proximal influences on their DFA [33]. A proliferation of research supports a positive correlation between children’s DFA and their parents’ DFA or unfavourable attitudes towards dentistry [3]. Our findings unveiled another facet of parental influence on children’s DFA. To the young girl who demonstrated a cluster of non-compliance, aggression, and destructive behaviors, her father’s careless joking statement, “the dentist would pull your teeth”, obviously cast a terrifying image of a dentist, petrified the child, deterred her attempt to cope, and rendered all the efforts of the dental team in vain. Information-giving is an inherent part of child-rearing and is carried out by parents in an almost unceasing fashion [26]. An interesting experiment demonstrated that parents’ threatening narratives about a friendly animal instilled high levels of fear in children [34]. This evidence illustrates how information from parents may shape their offspring’s view of the world. Thoughtful words from a sensitive parent could be a precious resource for the child to overcome his/her excessive fear and set the right expectation for the dental visit. Beyond family impact, adolescents are vulnerable to peer influence, which could be a significant source of their DFA. Although it is impossible to isolate teenagers from negative information from their peers, professionals should make parents aware of and sensitive to the potential influence of the information peers impart, so that parents can stand a better chance of protecting their offspring from developing DFA.

### Methodological Considerations and Limitations

Our findings can be better understood if the strengths and limitations of this study are recognized. To ensure the authenticity of our report and reduce the chances of exaggerated or biased contents, we excluded videos that were solely for entertainment or advertisement purposes. All videos analyzed in this study thus portrayed genuine experiences, feelings, or views of members of the public. A common concern in studies using social media that is applicable to this study is that they can only capture the views of people who are willing to share their personal feelings in the public forum. Parental control and safety precautions may deter some young children from uploading videos to YouTube. Therefore, it is not expected that the YouTube testimonials reported in this study represent all public opinions. Instead, they are better viewed as a supplement to information solicited from other channels for a more complete picture on DFA. Furthermore, not all age groups are equally attached to social media. Analysis on the YouTube profile of users showed that teens and young adults occupy the biggest proportions of users, while other age groups may be underrepresented [35]. This, however, should not have cast a negative impact on this particular study, since our expected informants were young adults (parents of young children) and teens (adolescents).

Our findings support the value of infodemiological studies using Internet social media to gain a better understanding of health issues [36]. Our study adopted an inductive method, in which themes emerge from data, rather than a deductive method, where themes are hypothesized based on theories and assumptions [11]. Inductive method, by its nature, is open-ended and exploratory, allowing us to discover an unrestricted range of public perspectives without being trapped within the boundary of professionals’ assumptions. Our approach of including a layperson with no formal dental background in the review panel may have contributed to obtaining accurate interpretations from the public’s perspective. The active participation of this layperson helped to avoid the pitfalls of professionals’ presumptions in interpreting at least 6 videos.

In this study, we included all eligible YouTube videos into the analysis, rather than drawing a sample from the YouTube platform. However, relevant videos on DFA of children and adolescents appeared in a relatively small volume (N=27). This may be due to children and adolescents’ limited ability in expressing themselves, which is a common concern for research in this age group. YouTube provides a channel for obtaining precious information from this often inaccessible group and their parents so that their voices can be heard and their feelings can be captured. The limited number of videos did not allow for data saturation in our analysis. Nevertheless, most of the themes emerged repetitively from the videos, supporting the relevance of these themes to children and adolescents.

### Conclusions

This qualitative study suggests that DFA in children and adolescents has multifaceted manifestations, impacts, and origins. Some of the themes only become apparent when using Internet social media. The novel and previously neglected themes emerged in this study can be attributed to the free sharing platform provided by YouTube, the candid in-depth testimonials in the videos, and the utilization of qualitative analysis, which allows the interpretation of the deep meanings of the informants. Our findings attached real life narratives to some of our existing knowledge on DFA and unveiled some missing pieces of the puzzles, which could be corroborated through further studies incorporating in-depth interviews with patients and parents.

The profound impacts of DFA on children and adolescents reinforce the idea that managing DFA should be a starting point in patient management. In light of its diverse origins, DFA could be better prevented and intercepted through coordinated efforts of dentists, dental auxiliaries, pediatric patients, and their parents. Thoughtful approaches before, during, and after the dental visit contribute in one way or another to a pleasant and productive dental experience. Successful DFA management not only paves the road to satisfactory clinical outcome and better oral health, but also builds confidence in pediatric patients and may help them regulate their emotions while facing other challenges in life.

## Abbreviations

DFA: dental fear and anxiety

## Multimedia Appendix 1

List of videos included in the study.
