# Supplementary material for: Dental Fear and Anxiety in Children and Adolescents: Qualitative Study Using YouTube
Source: J Med Internet Res. 2013 Feb 22;15(2):e29. doi: 10.2196/jmir.2290 (PMC3636260; doi:10.2196/jmir.2290)
Supplement: Supplementary file 1 [file jmir_v15i2e29_app1.pdf]

Mutlimedia Appendix. List of videos

Video number	Title	URL address
1	Dental care program for children and adults with Autism	http://www.youtube.com/watch?v=artQFqd6osQ
2	Child overcomes fear of dentist - East Bentleigh Dental Group Melbourne	http://www.youtube.com/watch?v=PiU2oQ52MTk
3	Tooth tales presents my first laser visit	http://www.youtube.com/watch?v=OvZgTD42RjU
4	Excellent childrens dentist on the Gold Coast -Ben Swindley Dental	http://www.youtube.com/watch?v=ZqHpzACVSqU
5	John explains why he trusts his familys' dental care with Ben Swindley Dental	http://www.youtube.com/watch?v=dea6PScclo0
6	End to fear Of the dentist?	http://www.youtube.com/watch?v=rDqLs0kHxNE
7	Elisa's first dental injection, the second attempt	http://www.youtube.com/watch?v=lt_C6TZSQVg
8	Child Dentistry - Cleaning out a cavity with the laser	http://www.youtube.com/watch?v=Kt6iaTeubB4
9	As easy as 1-2-3 - Dr. Neda Oromchian - General ...	http://www.youtube.com/watch?v=2wptDzphvFE
10	Smile Island Pediatric and Adult Dental Group Sacramento	http://www.youtube.com/watch?v=lxP1RdU3Zgg
11	Dr. Phillip Katz - Dentist in Ohio - Advanced Dentistry & Implants	http://www.youtube.com/watch?v=mbhAPpqRYHU
12	Laughing gas, Nitrous oxide gas, San Jose Child...	http://www.youtube.com/watch?v=ZuMeoZEDkEM
13	David after dentist	http://www.youtube.com/watch?v=txqiwrbYGrs
14	Part 1, pdd-nos 6-year-old boy visits DENTIST englewood ...	http://www.youtube.com/watch?v=pHIHviYPlKg
15	The discovery of the treadmill: Part one/braces	http://www.youtube.com/watch?v=roXwU65SKPQ
16	Actual dentist visit 1	http://www.youtube.com/watch?v=mQtOa_ItjVM&playnext=1&list=PLAEA42891825AD74F&index=28
17	Dentist-something in my mouth.3GP	http://www.youtube.com/watch?v=BFuHRNaSwic
18	Why I chose to combine dental visits and air travel	http://www.youtube.com/watch?v=sqqnvLweSY4
19	Niamh Jones talks to Brighton Dentist	http://www.youtube.com/watch?v=Y3LSGiDlEZ4
20	Dental anxiety mom and child; A dental success	http://www.youtube.com/watch?

	story	v=Br6nOtSAZnU
21	Mom and young daughter: Going to the dentist is a pleasant experience	http://www.youtube.com/watch?v=h_NBqcz653k
22	Phoenix Dentist	http://www.youtube.com/watch?v=fp0GetNFeHU
23	Got my wisdom teeth pulled out!!!!! yay!!! ...	http://www.youtube.com/watch?v=F2wF5RKhCNc
24	My dentist appointment from hell 2	http://www.youtube.com/watch?v=utYz6uO5yPU
25	Dr. Phillip Katz - Dentist in Ohio - Advanced Dentistry & Implants	http://www.youtube.com/watch?v=2pswBxXAhng
26	Cosmetic Dentistry Romeoville IL	http://www.youtube.com/watch?v=ypw1tbpXvpQ
27	Florissant Dental Services.mp4	http://www.youtube.com/watch?v=pCWlCN3lMPE&NR=1
